# Liver regeneration and fibrosis after inflammation

**DOI:** 10.1186/s41232-016-0025-2

**Published:** 2016-10-18

**Authors:** Minoru Tanaka, Atsushi Miyajima

**Affiliations:** 1 Department of Regenerative Medicine, Research Institute, National Center for Global Health and Medicine, Tokyo, Japan; 2 Institute of Molecular and Cellular Biosciences, The University of Tokyo, Tokyo, Japan

**Keywords:** Fibrosis, Hepatic stellate cell, Liver sinusoidal endothelial cell, Liver progenitor cell

## Abstract

The liver is a unique organ with an extraordinary capacity to regenerate upon various injuries. In acute and transient liver injury by insults such as chemical hepatotoxins, the liver in rodents returns to the original architecture by proliferation and remodeling of the remaining cells within a week. In contrast, chronic liver inflammation due to various etiologies, e.g., virus infection and metabolic and immune disorders, results in liver fibrosis, often leading to cirrhosis and carcinogenesis. In both acute and chronic inflammation, a variety of immune and non-immune cells in the liver is involved in the processes resulting in either regeneration or fibrosis. In addition, chronic hepatitis often accompanies proliferation of atypical biliary cells, also known as liver progenitor cells or oval cells. Although the origin of liver progenitor cells and its contribution to hepatic repair is still under intense debate, recent studies have revealed a regulatory role for immune cells in progenitor proliferation and differentiation. In this review, we summarize recent studies on liver regeneration and fibrosis in the viewpoint of inflammation.

## Background

**Fig. 1:**
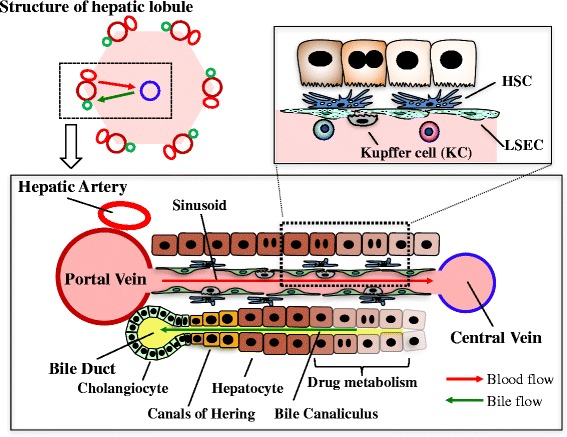
Schematic overview of the hepatic lobule. Blood flows into the liver from the portal vein and the hepatic artery toward the central vein through the sinusoid surrounded by fenestrated liver sinusoidal endothelial cells (LSECs). Bile produced by hepatocytes is collected into the bile ducts via the bile canaliculi surrounded by the apical membrane of hepatocytes. Kupffer cells (KC), resident macrophages in the liver, are located at the luminal side of the sinusoids, while hepatic stellate cells (HSCs) are positioned in close proximity to LSECs. The canals of Hering is the joint between hepatocytes and the bile ducts

The liver is a central organ for homeostasis and carries out a wide variety of functions, including metabolism, glycogen storage, drug detoxification, production of various serum proteins, and bile secretion. Most of those liver functions are carried out by hepatocytes, the liver parenchymal cells, which account for approximately 60 % of total liver cells and 80 % of the total liver volume. Hepatocytes are highly polarized epithelial cells and form cords (Fig. 1). Their basolateral surfaces face the sinusoid, a unique form of capillary in the liver, which consists of fenestrated liver sinusoidal endothelial cells (LSECs) and hepatic stellate cells (HSCs). Tight junctions formed between hepatocytes create a canaliculus surrounded by the apical membrane of neighboring hepatocytes. Bile secreted from hepatocytes is exported sequentially through the bile canaliculi, intrahepatic bile ducts, extrahepatic bile ducts, and finally into the duodenum. The bile duct is formed by another type of epithelial cell, biliary epithelial cell (BEC), also known as cholangiocyte. Hepatocyte and BEC are derived from a common progenitor, “hepatoblast,” during development [1]. In the similar context of liver progenitors, the adult liver also harbors a specialized type of cells which proliferates clonally in vitro and gives rise to hepatocyte and BEC depending on culture conditions [2, 3]. It has been believed that such a tissue stem cell-like progenitor contributes to hepatic repair in a case of emergency, e.g., severe or chronic liver injury. However, whether and where stem cells exist in the adult liver is still under debate.

Historically, the regenerative capacity of the liver is well known, and the mechanisms underlying liver regeneration have been investigated for many years. In 1931, Higgins and Anderson developed an experimental model of liver regeneration, i.e., surgical removal of rat median and left lobes that correspond to two thirds of the total liver mass [4]. Since then, the two-thirds partial hepatectomy (PHx) has been used as a standard model for liver regeneration. In this model, the remnant liver lobes enlarge to compensate for the lost mass, which is known as compensatory hyperplasia. After decades of studies on the liver regeneration from two-thirds PHx, it was believed that one or two replications of the remaining hepatocytes should be empirically sufficient to recover the original mass and function. However, revisiting this old theme by using modern techniques revealed that “hypertrophy” of hepatocytes precedes proliferation and that hypertrophy and proliferation contribute almost equally to the recovery of liver mass [5]. While PHx is an excellent model to study the process of compensatory growth of the liver and provides useful information relevant to liver transplantation, it does not faithfully recapitulate repair processes in human pathological conditions of liver diseases caused by virus infection, metabolic and immune disorders, drug intoxication, and so on. Here, we describe the cellular basis of liver regeneration and fibrosis after inflammation in acute and chronic liver injuries.

## Main text

### Metabolic zonation, drug-induced acute liver injury, and regeneration

The functional liver unit consists of the hepatic lobule, which has a central vein and hexagonal or polygonal portal triads consisting of the portal vein, hepatic artery, and bile duct. The central vein is connected to portal triads via sinusoids that run through the hepatic plates. Although all hepatocytes are morphologically similar, their functions are quite diverse and determined by their location along the porto-central axis of the functional liver unit, the hepatic lobule. Periportal hepatocytes are specialized for oxidative liver functions such as gluconeogenesis, ß-oxidation of fatty acids, and cholesterol synthesis, while pericentral hepatocytes are more important for glycolysis, lipogenesis, and cytochrome P450-based drug detoxification. Metabolic zonation is formed by a Wnt/ß-catenin signaling gradient [6, 7]. A recent study revealed that LGR4/5 receptors and their cognate RSPO ligands potentiate Wnt/ß-catenin signaling and control liver zonation [8].

Centrilobular hepatocytes express cytochrome P450s (Cyps) abundantly, which metabolize alcohol and various chemical hepatotoxins such as acetaminophen, carbon tetrachloride (CCl_4_), and thioacetamide, to generate highly reactive free radicals that damage hepatocytes. A single administration of drugs such as CCl_4_ induces necrosis of hepatocytes and disorganization of sinusoids surrounding the central vein. Proliferation of hepatocytes starts within 24 h, peaks at around 48 h, and terminates by 72 h in mice [9]. Along with proliferation of hepatocytes, sinusoid remodeling occurs in the necrotic area. Prior to these responses, hepatocytes damaged by free radicals produce damage-associated molecular patterns (DAMPs) to induce inflammation, by which the activated non-parenchymal cells contribute to regeneration. The resident and the recruited inflammatory cells from the bone marrow play a crucial role in regeneration and remodeling at the damaged area. The activated Kupffer cell, a resident hepatic macrophage, secretes interleukin-6 (IL-6) that directly induces hepatic expression of multiple genes associated with acute phase proteins, cell-cycle, redox, and anti-apoptosis to facilitate the proliferation of remnant hepatocytes [9–11]. HSCs and LSECs also play crucial roles in the proliferation of hepatocyte and sinusoidal remodeling after liver injury. The HSCs stimulated by inflammation contribute to the initiation of liver regeneration by secreting hepatocyte growth factor (HGF). In addition, the activated HSCs start to produce extracellular matrix (ECM) including collagens to fix the architecture of injured tissue in a similar manner to the process of wound healing [12, 13]. The ECM serves as a scaffold for the proliferation of hepatocytes and maintains the mechanical stability in the damaged region. The LSECs activated by acute inflammation also secrete HGF and Wnt2 to promote liver regeneration [14]. We have reported that Sema3e produced by damaged hepatocytes induces contraction of LSECs, which supports the activation of HSCs and the infiltration of leukocytes into the damaged area [15]. Given that insult of the liver is transient, these cells activated by inflammation will be eventually settled, followed by the resolution of ECM and revascularization. Thus, activation of non-parenchymal cells in the injured area and proliferation of undamaged hepatocytes must be well orchestrated to restore the original mass, functions, and structure of the liver in acute inflammation.

### Chronic liver injury and fibrosis

Chronic inflammation is an immune response that persists for months, in which inflammation and tissue remodeling and repair processes occur simultaneously. It can be induced by a number of different insults including hepatitis virus infection, excessive alcohol intake, autoimmune reactions, toxins, and metabolic disorders. However, regardless of etiology, chronic inflammation induces fibrosis that eventually leads to cirrhosis and hepatocellular carcinoma. In chronic hepatitis, activated HSCs become myofibroblasts and play a dominant role in fibrosis by producing a large amount of collagen. In addition, upregulation of a tissue inhibitor of metalloproteinases-1 (TIMP-1) in the fibrotic liver contribute to collagen deposition by inhibiting the resolution of ECM. Persistent production of growth factors for HSCs, fibrogenic cytokines, and chemokines by various types of liver cells are involved in fibrogenesis in chronic inflammation. Among those, TGF-ß produced by immune cells directly promotes fibrogenesis by inducing the transcription of type I and III collagen through the Smad signaling pathway [16]. IL-1ß and TNF-α do not induce HSC activation instead mediate the survival of activated HSCs and thereby contribute to liver fibrosis [17]. A recent study has revealed the implication of IL-33, an IL-1 family member cytokine in liver fibrosis. IL-33 secreted from damaged hepatocytes stimulates type 2 innate lymphoid cells (ILC2) to produce IL-13, which in turn promotes the activation of HSCs through STAT6 activation [18].

**Fig. 2:**
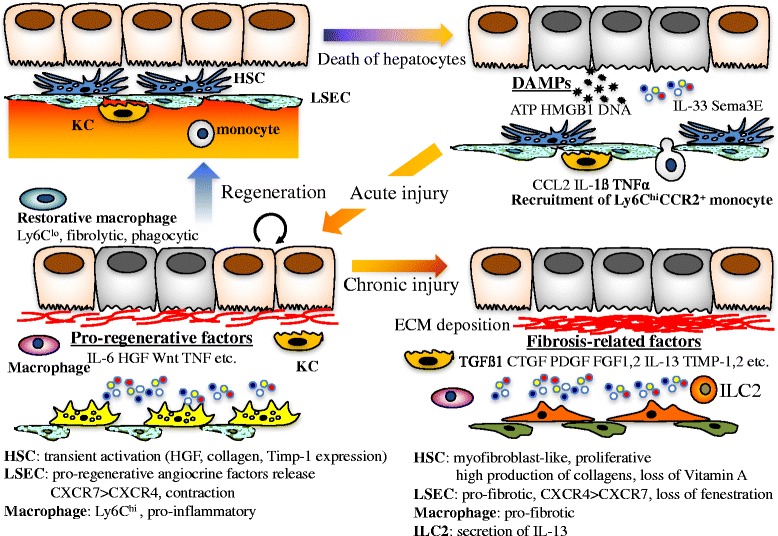
Phenotypic changes of non-parenchymal cells associated with liver regeneration or fibrosis after injury

Chemokines also play a role in liver fibrosis via non-immune cells as well as immune cells in the liver. Two types of receptors for CXCL12 (also called SDF1), CXCR4 and CXCR7, regulate a balance between regeneration and fibrosis after liver injury through the phenotypic change of hepatic vascular niche [14]. CXCR4 and CXCR7 are differentially expressed in LSECs depending on the condition of the damaged liver, and CXCR7 upregulation after acute injury contributes to liver regeneration by deploying pro-regenerative factors such as Wnt2 and HGF through the induction of transcription factor Id1. In contrast, constitutive FGFR1 signaling in LSEC under chronic hepatitis induces the predominance of CXCR4 over CXCR7 by augmenting CXCR4 expression, leading to a shift from pro-regenerative vascular niche to pro-fibrotic phenotype accompanied by the proliferation of activated HSCs. On the other hand, CCL2, also called MCP-1 secreted from Kupffer cell and HSC, contributes to the recruitment of CCR2+ Ly6C+ monocytes into the liver. The recruited Ly6C^hi^ macrophages are pro-inflammatory and pro-fibrotic and produce IL-1ß, TNF-α, TGF-ß, and PDGF to induce the survival, activation, and proliferation of myofibroblasts [19–22]. As such, hepatic macrophages contribute to liver fibrogenesis, while they play a crucial role in the resolution of ECM [23]. Ly6C^lo^ restorative macrophages have been reported to exhibit pro-resolution phenotypes with increased expression of fibrinolytic matrix metalloproteinases (MMPs) including MMP9 and MMP12, phagocytosis-related genes, and growth factors [20]. Thus, after acute inflammation, the phenotypic switch of pro-inflammatory macrophages to restorative macrophages together with the disappearance of pro-fibrotic macrophages plays important roles in liver regeneration and ECM resorption. Thus, interactions among immune and non-immune cells in response to persistent inflammatory factors can be a fork toward hepatic regeneration or fibrosis in chronic hepatitis (Fig. 2).

### Liver stem/progenitor cells and ductular reaction

Hepatocytes have a long lifespan, and new hepatocytes are derived from pre-existing hepatocytes. Thus, unlike intestinal stem cells, the liver homeostasis does not seem to require a resident stem cell population. Also, in acute liver injury, because remnant hepatocytes proliferate to restore the lost cells, stem cells are not necessarily needed. However, in chronic liver injury, it has been believed that liver progenitor cells (LPCs) or oval cells contribute to liver regeneration. Fundamentally, LPCs are defined as bi-potential cells similar to fetal hepatoblast, which can differentiate to both hepatocytes and BECs [1]. Chronic liver injuries often accompany “ductular reaction,” which is histologically characterized as ectopic emergence and expansion of bile duct marker-positive cells around the portal vein. It has long been postulated that ductular reaction represents the activation of adult LPCs that may reside in the biliary tree or the canals of Hering, the junctional structure connecting hepatocytes and the bile ducts. The concept of LPCs has been a paradigm in liver regeneration upon chronic injury, and most studies have focused on whether and how LPCs can proliferate and differentiate to hepatocytes to replenish the lost functions of the liver. Considering that LPCs expand in the case of chronic hepatitis, LPCs are supposed to be activated in response to inflammation. In fact, implications of several inflammatory cytokines, such as tumor necrosis factor (TNF)-alpha, interleukin-6, and interferon-gamma, in LPC proliferation have been reported [24–26]. Among those factors, TNF-related WEAK inducer of apoptosis (TWEAK) and fibroblast growth factor 7 (FGF7) are of particular interest, as they are capable of inducing de novo activation of LPCs without inflammatory insults, suggesting that the cell-of-origin for LPCs is responsive to these extracellular signals [27, 28]. Other growth factors, such as HGF and EGF, have also been implicated in regulating proliferation and/or differentiation of LPCs [29, 30]. Notch signaling is well known to play a pivotal role in the differentiation of fetal hepatoblasts into BECs [31–34]. In line with this notion, Boulter et al. reported that Jagged 1, a Notch ligand expressed by activated myofibroblasts, promoted the specification of LPCs to BECs during biliary regeneration [35]. Notably, macrophages engulfing hepatocyte debris expressed Wnt3a, which enhances canonical Wnt signaling and opposes Notch signaling in LPCs to promote their specification to hepatocytes during liver regeneration. Thus, LPCs are apparently a “facultative” stem/progenitor cell population that emerges around the portal vein for regeneration, depending on the microenvironment generated by chronic inflammation.

### A controversy issue on the role of LPC in regeneration

In sharp contrast to LPCs around the portal vein, Wang et al. identified a population of proliferating and self-renewing cells adjacent to the central vein by lineage tracing using the Wnt-responsive gene Axin2 in mice [36]. These pericentral cells expressed the early liver progenitor marker Tbx3, are diploid, and thereby differ from mature hepatocytes, which are mostly polyploid. Adjacent central vein endothelial cells provide Wnt signals that maintain such pericentral cells, thereby constituting the niche. The descendants of pericentral cells differentiate into Tbx3-negative polyploid hepatocytes and can replace all hepatocytes along the liver lobule during homeostatic renewal, although their contribution to hepatic repair after injury remains unknown. However, a more recent study showed that LGR4^+^ hepatocytes throughout the lobule contribute to liver homeostasis without zonal dominance, contradictory to the pericentral stem cell [8]. Furthermore, Font-Burgada et al. showed that there are a subset of periportal hepatocytes, “hybrid hepatocytes,” that express low levels of Sox9 and some bile duct-enriched genes, and it has been claimed that hybrid hepatocytes are the cells that primarily mediate liver injury repair [37].

In contrast, many recent studies employing genetic lineage-tracing approaches in vivo have shown that LPCs and/or pre-existing BECs do not or rarely contribute to new hepatocytes in mouse models, thereby raising a doubt on the concept that LPCs serve as the backup for hepatocyte regeneration [38–40]. These apparently contradictory results regarding the origin of new hepatocytes in chronic liver injury may be due to the differences in injury models employed. If healthy hepatocytes remain in the injured liver, they proliferate to restore normal functions, but biliary-derived LPCs may give rise to new hepatocytes when most hepatocytes are severely damaged. For instance, hepatocyte-specific genetic deletion of E3 ubiquitin ligase Mdm2 induced hepatocytes to apoptosis, necrosis, and senescence in those cells. Under such severe condition, LPCs are activated to reconstitute functional liver [41].

Lineage-tracing experiments have significantly advanced our understating on LPC and ductular reaction, while the cell-of-origin for LPC is still under intense debate. Using newly established imaging approaches to capture three-dimensional (3D) tissue morphology in situ, we have recently reported that ductular reaction essentially represents the dynamic and adaptive changes of ductal cells maintaining duct-like structure and connection with the portal bile ducts [42]. Clonal tracing further revealed the heterogeneity of BECs in terms of proliferation activity in vivo and that BECs in the periphery proliferate in a stochastic manner [43]. While it remains to be shown whether there is a specific class of BEC that functions as LPC by producing hepatocytes, it should be noted that the BEC marker-positive cells that emerge in chronic liver injury, which have been considered as LPC, are connected to the bile ducts.

## Conclusions

Liver regeneration is a well coordinated process by hepatocytes and non-parenchymal cells. However, persistent inflammation in chronic hepatitis alters the well-ordered phenotypic changes of non-parenchymal cells and leads to an aberrant healing process, i.e., liver fibrosis. Along the progression of fibrosis, the replacement of the damaged tissue with ECM impairs the functions, flexible structure, and regeneration capacity of the liver. Although the most effective therapy for fibrosis to date is elimination of causative agents in earlier stages, it is insufficient to restore the cirrhotic liver to its original condition in many cases. Liver fibrogenesis is often accompanied by the emergence of LPCs, suggesting that fibrotic environment including activated myofibroblasts and immune cells may serve as a niche for proliferating LPCs. Further investigation of regulatory mechanisms underlying liver fibrosis and the role of LPCs in regeneration will help in developing therapeutic strategies to counter liver disease.

## Abbreviations

BEC, biliary epithelial cell; DAMPs, damage-associated molecular patterns; ECM, extracellular matrix; HSC, hepatic stellate cell; LPC, liver progenitor cell; LSEC, liver sinusoidal endothelial cell; MMP, matrix metalloproteinase; TIMP-1, tissue inhibitor of metalloproteinases-1; TWEAK, TNF-related weak inducer of apoptosis
